# Ultrafast genome-wide inference of pairwise coalescence times

**DOI:** 10.1101/gr.277665.123

**Published:** 2023-07

**Authors:** Regev Schweiger, Richard Durbin

**Affiliations:** Department of Genetics, University of Cambridge, Cambridge CB2 1TN, United Kingdom

## Abstract

The pairwise sequentially Markovian coalescent (PSMC) algorithm and its extensions infer the coalescence time of two homologous chromosomes at each genomic position. This inference is used in reconstructing demographic histories, detecting selection signatures, studying genome-wide associations, constructing ancestral recombination graphs, and more. Inference of coalescence times between each pair of haplotypes in a large data set is of great interest, as they may provide rich information about the population structure and history of the sample. Here, we introduce a new method, *Gamma-SMC*, which is more than 10 times faster than current methods. To obtain this speed-up, we represent the posterior coalescence time distributions succinctly as a gamma distribution with just two parameters; in contrast, PSMC and its extensions hold these in a vector over discrete intervals of time. Thus, Gamma-SMC has constant time-complexity per site, without dependence on the number of discrete time states. Additionally, because of this continuous representation, our method is able to infer times spanning many orders of magnitude and, as such, is robust to parameter misspecification. We describe how this approach works, show its performance on simulated and real data, and illustrate its use in studying recent positive selection in the 1000 Genomes Project data set.

Each of an individual's two alleles, at each genomic position, is inherited from a chain of ancestors going back in time. These two chains must eventually coalesce at an identical common ancestor. The length of these chains, measured by the (scaled) number of generations until coalescence, is called the *coalescence time*, or the *time to the most recent common ancestor* (TMRCA). Coalescence times reflect the genealogical and genetic history of a sample and, therefore, have been used in many population and statistical genetic analyses, such as reconstructing demographic histories (Li and Durbin 2011; Schiffels and Durbin 2014; Terhorst et al. 2017; Wang et al. 2020), detecting selection signatures (Palamara et al. 2018; Nait Saada et al. 2020; Hejase et al. 2022), studying genome-wide associations (Minichiello and Durbin 2006; Zhang et al. 2023), imputing genotypes (Zhang et al. 2023), and more.

As genomic data sets grow larger, inference of coalescence times between each pair of haplotypes in a large data set is of great interest, as they may provide rich information about the population structure and history of the sample in resolution impossible to attain otherwise. Moreover, they may further be used to construct an ancestral recombination graph (ARG) (Griffiths and Marjoram 1996), a sequence of local genealogies that documents the genealogical history of each locus across the sample.

Coalescence times along two homologous chromosomes can be well approximated with a Markov chain using the sequentially Markovian coalescent (SMC) framework (McVean and Cardin 2005; Marjoram and Wall 2006). This framework underlies the PSMC (Li and Durbin 2011) method, which estimates a posterior distribution of the TMRCAs, as well as infers a history of change in the effective population size over time. PSMC and its successors are based on a hidden Markov model (HMM), in which the hidden states along the genome correspond to pairwise TMRCAs, and the emissions are the observed diploid sequence. Although PSMC was designed to work on a single pair of sequences, follow-up methods have been developed to scale to increasingly larger sample sizes (for a review, see Mather et al. 2020). The multiple sequentially Markovian coalescent (MSMC) (Schiffels and Durbin 2014) analyzes the genomes of multiple individuals, focusing on the first coalescence between any two individuals, but is computationally prohibitive and can only be applied to up to eight samples. MSMC2 (Wang et al. 2020) uses a composite likelihood approach instead but is still limited in sample size. The diCal method (Harris et al. 2014) allows decoding coalescence times in linear time with respect to the state space of the HMM instead of the quadratic complexity in PSMC, MSMC, and MSMC2. The SMC++ method (Terhorst et al. 2017) allows incorporating allele frequencies from hundreds of unphased genomes to decode coalescence times more accurately for a focal pair of haplotypes. The XSMC method modifies the SMC model, avoiding discretizing the HMM state space and leading to exact and efficient inference of coalescence times under the revised model (Ki and Terhorst 2020). Finally, the recent ASMC method (Palamara et al. 2018) incorporates computational and algorithmic improvements that reduce running times by two to four orders of magnitude and extends the approach to work on genotyping arrays. Despite these advances, further scaling TMRCA inference is still desirable.

To facilitate such large-scale analyses, we introduce a new method, Gamma-SMC, which is 14–20 times faster than current methods. To obtain this speed-up, we represent the posterior coalescence time distributions succinctly as a gamma distribution with just two parameters rather than as a vector over discrete intervals of time as in PSMC and its extensions. Thus, Gamma-SMC has constant time-complexity per site, without dependence on a number of discrete time states. Additionally, because of this continuous representation, our method does not depend on a choice of discretization scheme, which allows it to avoid discretization and boundary effects. This allows it to be robust to misspecification of the mutation rate, which can artificially inflate or deflate the scale of coalescence times, and therefore, it is also able to reliably infer times spanning several orders of magnitude. We describe how this approach works and illustrate its performance on simulated and real data. Finally, as an example of its utility, we show how the estimated coalescence times can be used to scan for sites under recent positive selection in the human genome.

## Results

### Overview of the Gamma-SMC method

Like its predecessors (Li and Durbin 2011; Terhorst et al. 2017; Palamara et al. 2018; Schiffels and Wang 2020), Gamma-SMC is based on an HMM with transition probabilities derived from the SMC framework, with hidden states indicating pairwise TMRCAs and the observed diploid sequence serving as emissions. However, standard HMMs require a discrete hidden state space, whereas coalescence time is naturally continuous. Therefore, current SMC-based methods approximate the full model by discretizing time into a number of intervals (e.g., *T* = 20, 32, or 64) and so have at least *O*(*T*) computational complexity per step.

#### Avoiding time discretization with a gamma approximation

We now describe how Gamma-SMC avoids time discretization. We focus here on the forward algorithm of the HMM, but similar arguments hold for the backward algorithm and for combining both forward and backward passes (Methods). The forward algorithm iteratively updates the posterior distribution of the hidden state, conditional on the observations seen so far (Rabiner 1989; Bishop and Nasrabadi 2006). That is, it measures and updates our uncertainty of the coalescence time at a locus, given the sequence up until that locus. Instead of holding this distribution as a vector as in current methods, we observed, as others have (Albers and McVean 2020), that the SMC posteriors are very well approximated by a gamma distribution. If we constrain the posterior to be a member of the family Γ(*α*, *β*), then the hidden state is described by only two parameters (*α* and *β*) per step. Analogs of all standard HMM algorithms have been derived for continuous-state HMMs (CS-HMMs) (Ainsleigh 2001), and in principle, this leads to *O*(1) time-complexity per step, compared with *O*(*T*) or even *O*(*T*^2^) by current methods.

The motivation for using the gamma distribution is threefold. First, the gamma distribution is a rich family, well-suited to model positive, unimodal, and continuous distributions, with only two parameters. Second, the gamma distribution is conjugate to the Poisson distribution, which underlies the emission probability in the HMM. This was discussed by Hein et al. (2004), equation 3.45, and was also used by Albers and McVean (2020). This means that, without recombination and with the natural exponential prior on coalescence time, the forward densities should be exactly gamma. It is therefore reasonable that with a small recombination probability per step, the gamma shape may still approximate the true density well. Finally, an empirical study of forward densities of PSMC shows a gamma distribution approximation often well approximates the true density (Supplemental Methods; Supplemental Fig. S1).

To support HMM operations, we need to describe how to update the forward density from one step to the next. As mentioned above, following recombination, the forward density in the next step need not be gamma, even if it was gamma at the previous step. For small recombination rates, consecutive forward densities will be very similar, so we can likely approximate the next step with gamma as well. The forward algorithm alternates between two steps: (1) multiplying the current forward density by the transition density to account for additional uncertainty arising from a possible recombination event, and (2) updating the new density using the emission probability to account for the additional information from the new observation. We explain each in turn.

For the emission step, we can exploit the conjugacy between Poisson and gamma to derive a simple update rule for the *α*, *β* parameters. Denote the *i* + 1-th observation by *y* ∈ {0, 1}, indicating if this is a heterozygous (het) site, where the sequences differ, or a homozygous (hom) site, identical in both sequences. If the posterior density of the TMRCA at time *i* + 1, conditional on the first *i* observations, is Γ(*α*, *β*), then the forward density in step *i* + 1 is simply Γ(*α* + *y*, *β* + *θ*), where *θ* = 4*N*_*e* _*μ* is the scaled mutation rate.

For the transition step, we derived a closed-form expression of the coalescence time at step *i* + 1, given that the forward density at step *i* is gamma (Supplemental Methods). This expression is not in the form of a gamma distribution, but for a small recombination rate, it is expected to be close. Therefore, for each possible Γ(*α*, *β*) distribution, we find a new approximate subsequent Γ(*α*′, *β*′) distribution. The mapping $F:(\alpha ,\beta )\to (\alpha^{\prime },\beta^{\prime })$ may be viewed as a flow field, or a two-dimensional vector field, describing a dynamical system that develops along the genome. We evaluated this flow field over a log-spaced grid (Methods) and use bilinear interpolation for off-grid values, ensuring that the interpolated posterior meets certain consistency requirements (for details, see Supplemental Methods). Importantly, although calculating this flow field is computationally expensive, it needs only to be calculated once.

#### Locus skipping

As in other SMC methods, we preprocess the genome into segments. Each segment ends with either a het site or a site defined by the user as a target for inference or when a maximal length (e.g., 10 kbp) is reached. Within each segment are only hom sites (for discussion of missing sites, see Methods). We calculate in advance the result of repeatedly applying transition and emission steps with hom sites, up until maximal segment length, and cache these results. Then, the forward pass can effectively skip stretches of hom sites and be evaluated only at the segment ends. This allows for additional substantial speed-up.

#### Comparison with XSMC

The XSMC method (Ki and Terhorst 2020) departs from the SMC model by using the renewal approximation (Carmi et al. 2014), which assumes that following a recombination event, the new TMRCA is drawn independently of the previous TMRCA. Similar to Gamma-SMC, this revised model uses a continuous state space. It also allows for exact analytic solutions for pointwise posterior decoding of TMRCAs, for sampling paths of coalescence times from the posterior path distribution conditional on observations, and for calculating the maximum a posteriori (MAP) hidden state path.

Both XSMC and Gamma-SMC use a CS-HMM that avoids time discretization and use gamma distributions in their solutions. However, the two methods make different key approximations. XSMC uses the renewal approximation, which results in the XSMC forward and backward distributions being a mixture of gamma distributions, with the full posterior conditional density being a ratio of a product of gamma mixtures and another gamma distribution. Unlike XSMC, Gamma-SMC uses the full SMC model, therefore making use of the correlations between adjacent TMRCAs in inference. However, it approximates the forward, backward, and posterior densities to be a single gamma distribution. Finally, Gamma-SMC is designed for computationally efficiency, whereas XSMC focuses more on exactness of solution. Below, we benchmark the performance of XSMC in running time and accuracy.

### Benchmarks

#### Accuracy

**Figure 1.**
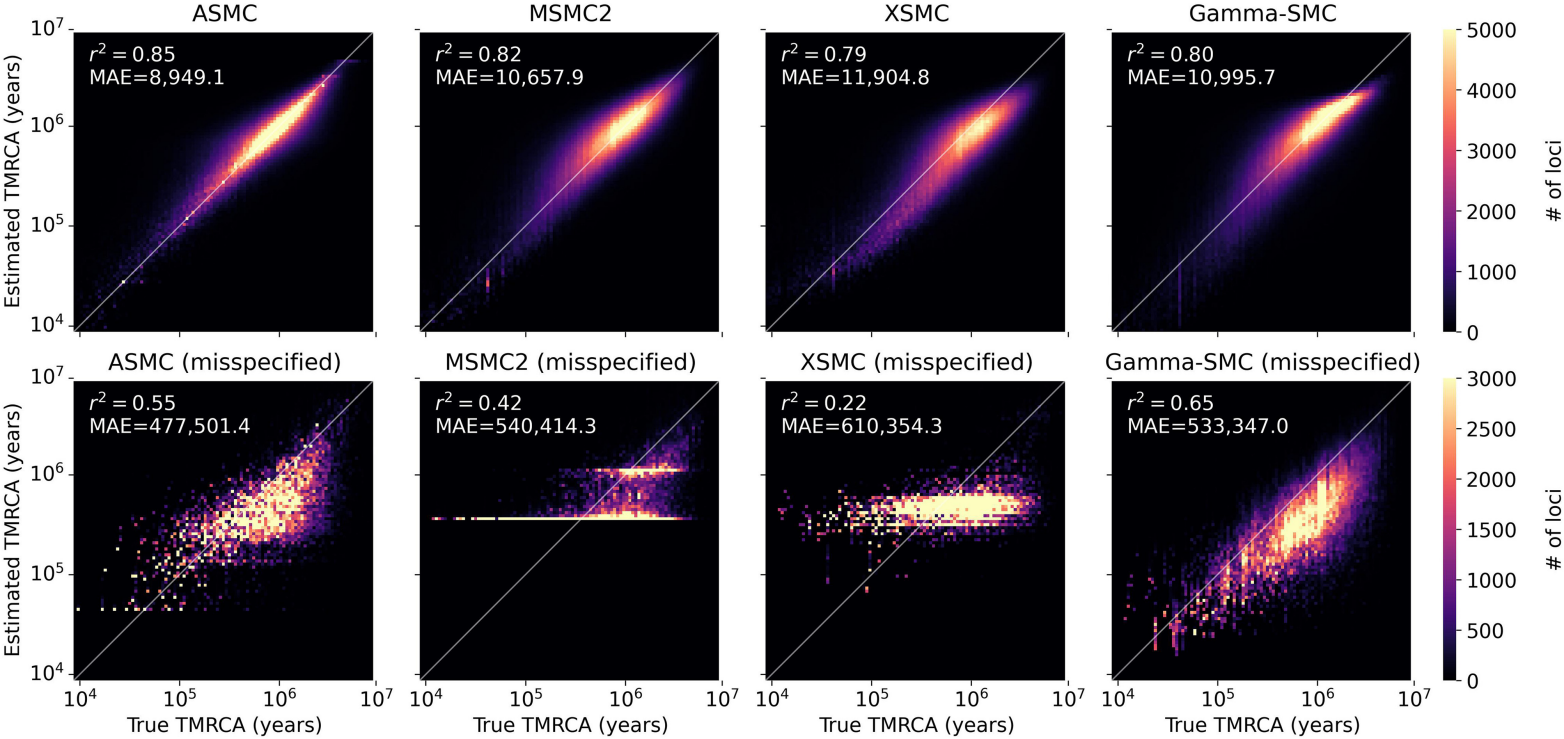
A comparison of the true TMRCA versus the estimated TMRCA (posterior mean) across a simulated genome. Shown for ASMC-seq, MSMC2, and XSMC versus Gamma-SMC when the correct population genetic parameters are given (*top*) and when parameter misspecification causes TMRCAs to be 100 times smaller than expected (*bottom*). Pearson's *r*^2^ and the mean absolute error (MAE; in generations) are shown.

We conducted a simulation study to evaluate the accuracy of Gamma-SMC compared with current SMC-based methods. We simulated a whole genome using realistic population genetic parameters (Methods) and used ASMC-seq (Palamara et al. 2018), MSMC2 (Wang et al. 2020), XSMC (Ki and Terhorst 2020), and Gamma-SMC to infer the posterior mean of the coalescence times. For XSMC, we sampled 100 paths from the posterior path distribution and took the median coalescence time every 1 kbp. When comparing the true TMRCA and its inferred posterior mean (Fig. 1), we conclude that all methods have similar inference accuracy, with ASMC-seq more accurate and with the approximation-based methods XSMC and Gamma-SMC less accurate. This is further confirmed by measuring Pearson's correlation *r*^2^ (ASMC-seq = 0.85, MSMC2 = 0.82, XSMC = 0.79, Gamma-SMC = 0.80) and the mean absolute error (in generations, ASMC-seq = 8949.1, MSMC2 = 10,657.9, XSMC = 11,904.8, Gamma-SMC = 10,995.7) between the true and inferred TMRCA. Although Gamma-SMC performance here is lower than that of the discretized HMM algorithms, as we will see below, it is good enough for further inference.

**Figure 2.**
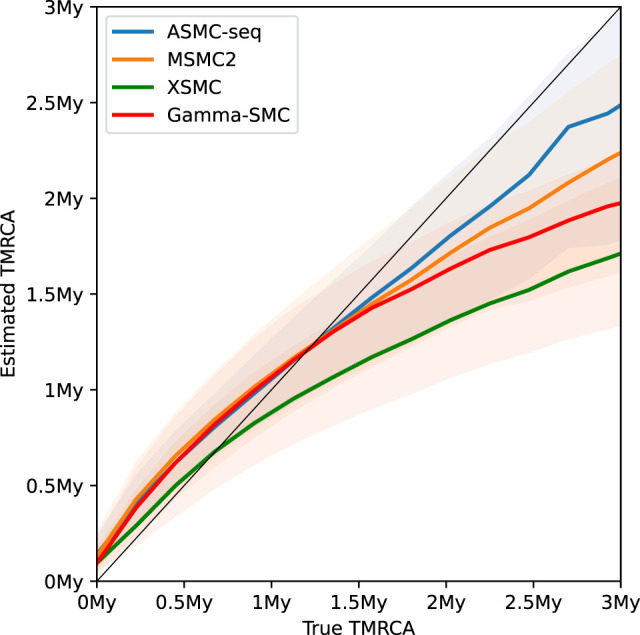
A study of TMRCA inference bias in Gamma-SMC versus MSMC2, XSMC, and ASMC-seq. The mean inferred TMRCA is shown as a function of the true TMRCA (confidence band, 25%–75% quantiles).

We next analyzed the distribution of the posterior mean TMRCA estimator across ASMC-seq, MSMC2, XSMC, and Gamma-SMC (Fig. 2). We note that, although all methods tend to regress to the mean as expected of posterior Bayesian estimators, overestimating low TMRCA values and underestimating high TMRCA values, mean Gamma-SMC and XSMC estimates are lower than mean ASMC-seq and MSMC2 estimates in each bin of true TMRCA, suggesting there might be a slight consistent bias that could be corrected.

#### Robustness to scale

Another drawback of the time discretization used by current methods is that inference resolution is determined by the discretization scheme chosen, so that it is complex if not impossible to reliably distinguish between TMRCAs in the same time interval. In particular, inference will be poor if the true TMRCA is very small; this may happen, for example, in a long run of homozygosity or with parameter misspecification.

To examine this, we simulated a diploid sequence and used ASMC-seq, MSMC2, XSMC, and Gamma-SMC to estimate TMRCAs, but in the simulation we used an effective population size of 150, effectively decreasing by a factor of 100 the true underlying TMRCAs relative to the tuning of the algorithms. When such small TMRCAs are estimated by MSMC2, the interval boundaries make inference coarse and imprecise, whereas with Gamma-SMC, inference is much less affected (Fig. 1). ASMC-seq inference is better than MSMC2 and XSMC but worse than Gamma-SMC (*r*^2^ of ASMC-seq = 0.55, MSMC2 = 0.42, XSMC = 0.22, Gamma-SMC = 0.65). We note that the effective range in Gamma-SMC is determined only by the grid values the flow field was precalculated on, such that arbitrarily small or large TMRCAs can be inferred if needed.

#### Demographic model

Gamma-SMC assumes an unrealistic constant-size population demographic model in its flow field construction. To test the effect of this assumption, we simulated a CEU human genome using the out-of-Africa model of Gutenkunst et al. (2009), as implemented by stdpopsim (Adrion et al. 2020), and inferred TMRCAs using ASMC-seq, MSMC2, XSMC, and Gamma-SMC (Supplemental Fig. S2). For ASMC-seq, we used the provided CEU demographic model in the inference, whereas other methods are not able to use a prior demographic model. We observed that all methods handle the demographic model discrepancies well and do not seem to have a consistent bias. They have a similar *r*^2^, with XSMC least correlated (*r*^2^ of ASMC-seq = 0.9, MSMC2 = 0.87, XSMC = 0.81, Gamma-SMC = 0.85). Similarly, the mean absolute error is lowest for ASMC-seq and highest for XSMC (ASMC-seq = 3685.2, MSMC2 = 4069.4, XSMC = 5019.5, Gamma-SMC = 4196.1). We conclude that Gamma-SMC is still helpful even in the face of some demographic model misspecification, although care must be taken when using absolute quantities, rather than relative estimates.

#### Speed

We next evaluated the running time of Gamma-SMC and compared it to the state-of-the-art method, ASMC-seq, as well as to MSMC2 and XSMC. The running time of Gamma-SMC in practice is affected by several factors, such as the number of segregating sites, patterns of missing data, and more. For a fair comparison, we used the example provided in the ASMC Git repository: 5086 segregating sites within 30 Mbp, across 150 diploids (300 haplotypes, 44,850 haplotype pairs), with no missing sites.

The XSMC software provides two options for inference: (1) calculating a MAP path and (2) sampling coalescence time paths from the posterior distribution, to further process them, for example, by taking the position-wise median. We benchmarked both the calculation of the XSMC MAP path and the drawing of a single path from the posterior, finding both to be at least an order of magnitude slower than Gamma-SMC, per diploid pair. We note that for accurate inference, many sample paths need to be drawn per pair (e.g., 10 or 100), further slowing down inference.

**Figure 3.**
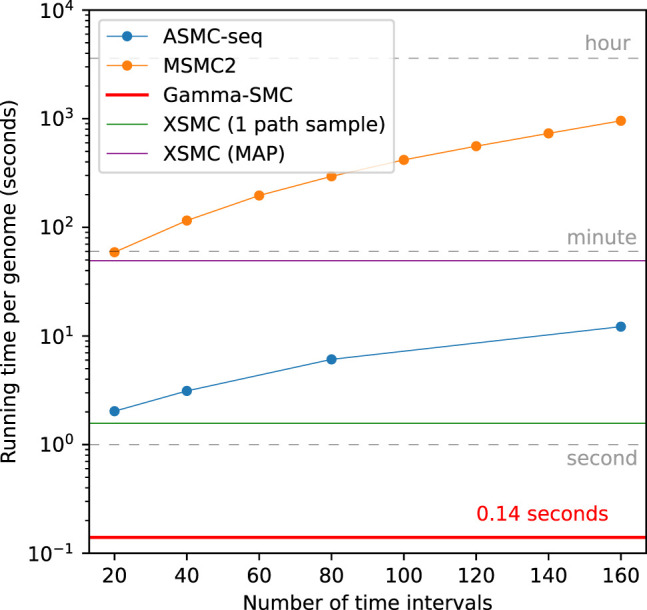
Running time of Gamma-SMC versus MSMC2, XSMC, and ASMC-seq for a whole genome (3.2 Gbp).

We observed that the running time of Gamma-SMC is at least 14× faster than ASMC-seq (Fig. 3), depending on the number of discrete TMRCA intervals used in ASMC-seq. For example, analysis of all pairs using *T* = 69 discrete time intervals, as is the smallest precomputed default provided by ASMC-seq, required 29 min for a single processor for ASMC-seq compared with ∼1 min for Gamma-SMC. Extrapolated to a genome size of 3.2 Gbp, Gamma-SMC can process a whole genome in ∼0.14 sec.

### Detecting selective sweeps

To further show the utility of Gamma-SMC, we show how it may be used to detect loci under recent positive selection. Positive selection at a locus causes a rapid rise in the frequency of the beneficial allele. As such, haplotypes with the beneficial allele will coalesce much faster than for neutral sites. When selection is recent, this causes an enrichment for recent coalescence times in the distribution of pairwise TMRCAs at positively selected loci. This skew can be used as a basis for a test for selection (Palamara et al. 2018; Stern et al. 2019; Hejase et al. 2022), by looking for genomic regions enriched for recent coalescence times. Here, we follow the approach introduced by Palamara et al. (2018) to show utility using the output of Gamma-SMC.

To assess this approach for positive selection detection, we first conducted a simulation study. We simulated regions of 10 Mbp, where the central positions have undergone a selective sweep. We varied the selection coefficient and fixed either the sample allele frequency or the mutation age (for details, see Methods). We compared our approach to several standard selection detection methods: the classical Tajima's D (Tajima 1989), iHS (Voight et al. 2006), and nSL (Ferrer-Admetlla et al. 2014). For Gamma-SMC, we simply computed the proportion of pairwise coalescence times below a threshold of *T* generations for several choices of *T*. We averaged the test statistics over several choices of window size around the focal locus. For each method and choice of window size, we calculated the true-positive rate (TPR), false-positive rate (FPR), and area under the curve (AUC) as a function of the test statistic. The full results are available at Supplemental Table 1.

We observed that for Gamma-SMC, the performance on average is similar between window sizes of 100 kbp or 10 kbp or having no window (just the focal site). For iHS and nSL, performance slowly deteriorates with window size. We also saw that when the sample allele frequency is high (0.9), Gamma-SMC performs significantly better than the other methods. The reason for this is that iHS and nSL are based on the contrast between patterns around the major and minor alleles, so that with a hard sweep, the minor allele is too rare for these tests to give useful information. In cases in which iHS or nSL do not apply to any position in the averaged window, we artificially set its statistic to a *Z*-score of −10 (corresponding to no selection detected). Conversely, Gamma-SMC will actually have more power when there are more pairs with the advantageous allele; for a representative example in which Gamma-SMC has better performance, see Supplemental Figure S3. For lower allele frequencies and for a range of mutation ages, we generally saw that Gamma-SMC is among the best of the methods considered here (usually alongside either iHS and nSL) across a range of parameters. The best-performing *T* varies with selection coefficient and mutation age, with 2000–4000 generations giving the best performance on average.

### Application to 1000 Genomes Project data

We applied Gamma-SMC to 503 individuals of five populations of European ancestry from the 1000 Genomes Project data set (Phase 3) (The 1000 Genomes Project Consortium 2015), assuming a scaled mutation rate of *θ* = 0.00075 and scaled recombination rate of *ρ* = 0.0006, corresponding to an unscaled mutation rate *μ* = 1.25 × 10^−8^, an unscaled recombination rate *r* = 10^−8^, and an effective population size of *N*_*e*_ = 15,000. We inferred the posterior distribution of the TMRCAs for each pair of the 1006 haplotypes, at evenly spaced intervals of 1 kbp across all chromosomes. Excluding data loading and writing results, this took a total of 120.5 CPU h for 505,515 haplotype pairs, computed in parallel across 7150 jobs. (At 0.85 sec per pair, this is slower than the value given above, essentially because of masking and pre- and postprocessing.)

**Figure 4.**
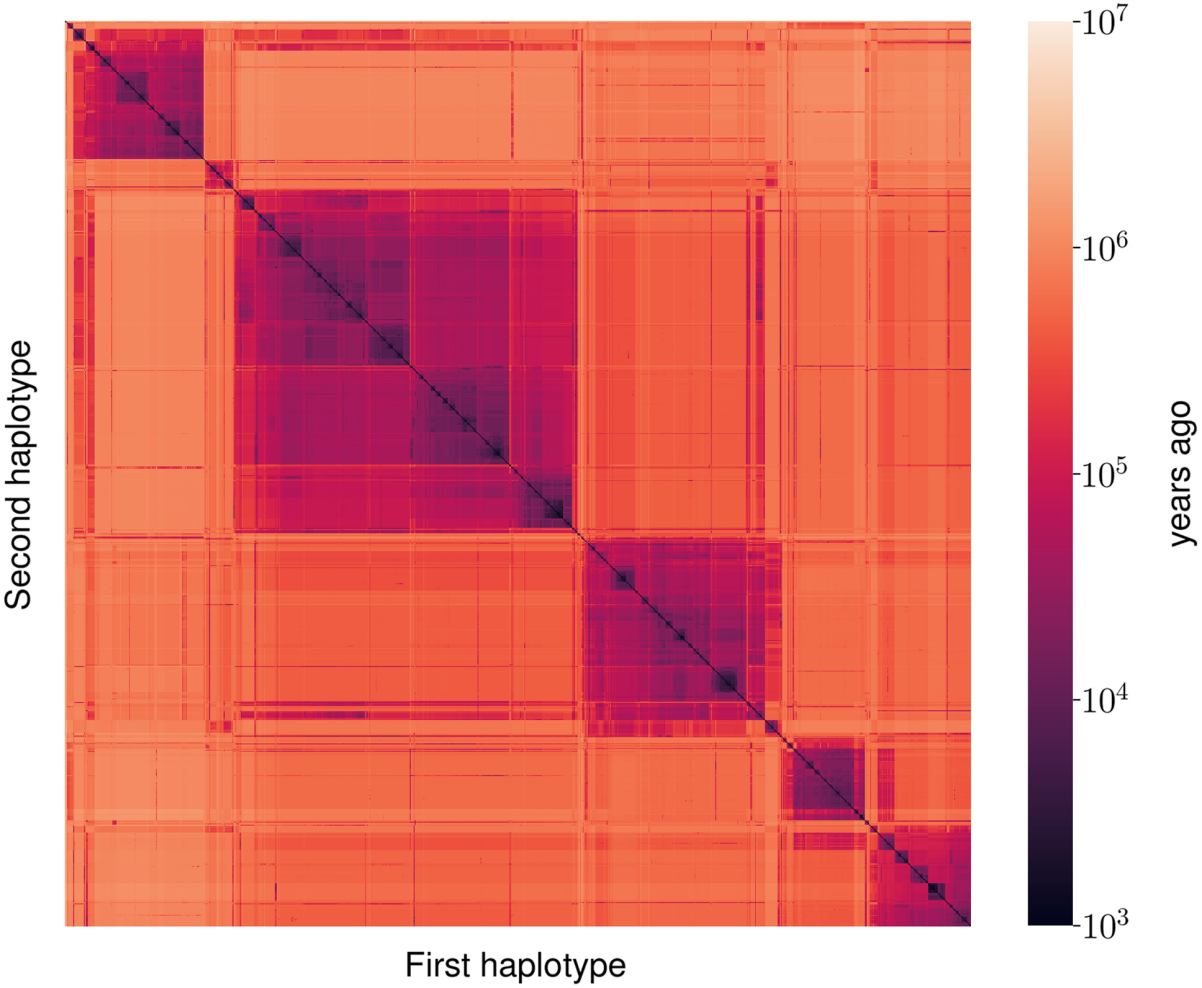
Pairwise coalescence time for all haplotype pairs of European individuals from the 1000 Genomes Project data, at Chr 2: 500,000. Coalescence times are estimated by taking posterior means, measured in years ago. Times were converted from generations to years using a generation time of 30 yr.

We illustrate the results by showing an example of inference at a single position (Fig. 4). We show a heatmap of the posterior mean TMRCA between each pair of haplotypes. The order of haplotypes is given by UPGMA hierarchical clustering. We note the clear hierarchical structure, corresponding to the coalescent tree at this position.

**Figure 5.**
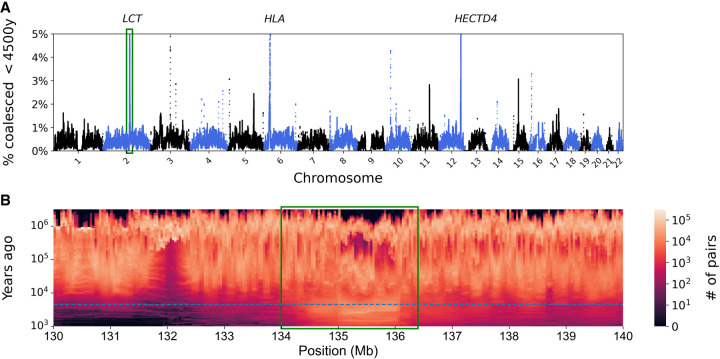
Scanning for recent positive selection. (*A*) A search for 100-kbp regions enriched for pairwise coalescence times more recent than 4500 yr ago. (*B*) A focus on the locus of the *LCT* gene (green rectangle), showing an enrichment of coalescence time in recent years (dashed line, 4500 yr ago; times were converted from generations to years using a generation time of 30 yr).

We then performed a selection scan by looking for genomic regions of size 100 kbp, where a large percentage of pairwise coalescences are estimated to have occurred in the past 4500 yr (Fig. 5A). We recover approximately 20 candidate loci, including, for example, *LCT* (Mathieson et al. 2015), the gene encoding the lactase enzyme, and the HLA region, both known to have been under recent positive selection in Europeans (Fig. 5B). The peak at Chromosome 12 falls inside *HECTD4*, which has recently been found to be under selection in Eurasians (Irving-Pease et al. 2022), although we note that the credible interval contains other genes.

## Discussion

We present Gamma-SMC, a method for efficiently inferring coalescence times from sequencing data. We showed that Gamma-SMC is at least an order of magnitude faster than current methods and that it can infer very small or very large TMRCAs accurately, owing to its continuous state space. Finally, we applied Gamma-SMC to 505,515 pairs of haploid genomes from the 1000 Genomes Project data and detected evidence for recent positive selection at multiple sites across the genome.

Although Gamma-SMC shares some similarities with XSMC, such as the use of gamma distributions to avoid discretization and to approximate the posterior coalescence distributions, there are distinct differences in the approximation techniques used by the two methods. We observed that both methods experience similar degradation in accuracy compared with MSMC2 and ASMC, which use the underlying SMC model without approximation, up to discretization. However, in practical terms, Gamma-SMC shows significantly faster performance, particularly when considering the multiple path samples required to reasonably approximate the posterior mean in XSMC. We believe there is potential to combine the strengths of both methods: One promising direction is to explore the incorporation of a mixture of gammas into Gamma-SMC, drawing on the benefits of both approaches.

We note several limitations of Gamma-SMC. As with other SMC-based methods, good quality phasing is required to apply Gamma-SMC across individuals. Although possible for large data sets, it may be harder to obtain for smaller ones. In addition, although the accuracy of Gamma-SMC is comparable to current methods, it could be further improved. Directions include incorporating genetic maps for inference, using more accurate demographic models when building the flow field, incorporating a post-hoc TMRCA normalization step (Zhang et al. 2023), and using the conditioned sample frequency spectrum (Terhorst et al. 2017; Palamara et al. 2018), which uses allele frequencies in inference. In terms of speed, GPUs may offer additional acceleration. Finally, Gamma-SMC currently works only on sequencing data. Extending Gamma-SMC to use the conditional Simonsen–Churchill model (Hobolth and Jensen 2014) would enable it to operate on SNP genotyping array data and is a promising direction for future research.

One exciting potential application of Gamma-SMC is in constructing an ARG. Indeed, the clear hierarchical structure evidenced in the pairwise posterior TMRCAs (Fig. 4) reflects the genealogical tree structure at a site that, when inferred along the genome, gives rise to the tree sequence representation of an ARG (Zhang et al. 2023). Gamma-SMC may improve branch placement and timing in the weaving step of ARG building methods such as ARGweaver (Rasmussen et al. 2014) and ARG-Needle (Zhang et al. 2023), as the continuous nature of the posterior distributions of coalescence times in Gamma-SMC may help resolve ambiguities present in discrete time approximations.

Although ARGweaver (Rasmussen et al. 2014) samples ARGs from a posterior distribution, it does not scale to larger sample sizes. Conversely, a disadvantage of the current scalable ARG building methods is that they only output a single ARG topology (although we note Relate [Speidel et al. 2019] samples from the posterior distribution of branch lengths). Further focusing on a particular locus, there is interest in sampling from the posterior distribution of trees at a single position, as this allows quantifying the uncertainty in both dating of coalescence events and the topology of the tree. Given pairwise coalescence posterior distributions from Gamma-SMC, it is possible to envision an approach that samples the first coalescence, infers the ancestral sequence at this coalescent event, and then reruns Gamma-SMC from this to each of the other sequences, iterating these steps in order to sample a local tree.

Previous methods also infer the history of population size of the sample, but inference is limited in recent times owing to the sparsity of recent coalescence events. However, the number of pairs in a population sample grows quadratically with its size, so that for a large sample, many more recent coalescence events are expected. Using this for demography inference is another promising avenue of research.

## Methods

### Gamma-SMC

We outline the structure of the full Gamma-SMC algorithm, while deferring most mathematical derivations to the Supplemental Methods. We denote by *μ* the unscaled mutation rate, in units of mutations per base pair per generation; by *N*_*e*_ the effective population size, in number of diploids; by *θ* = 4*N*_*e *_*μ* the scaled mutation rate; by *r* the unscaled recombination rate, in units of recombinations per base pair per generation; and *ρ* = 4*N*_*e*_*r* the scaled recombination rate.

#### Flow field

Let *f*_*α*,*β*_(*x*) be the probability density function (pdf) of the gamma function Γ(*α*, *β*). Let *p*(*t*|*s*) be the SMC transition distribution, given in the Supplemental Methods. Assuming that the forward density at time *i* is distributed as Γ(*α*, *β*), we define *p*_*α*,*β*_(*t*) to be the compound distribution $\;p_{\alpha ,\beta }(t):=\int p(t|s)\cdot \;f_{\alpha ,\beta }(s)ds$. Therefore, *p*_*α*,*β*_(*t*) defines the posterior distribution of the TMRCA at time *i* + 1, given that the forward density at time *i* is modeled as gamma. In the Supplemental Methods, we show how to evaluate this compound distribution analytically in the case of a constant population size and approximate it with another gamma distribution Γ(*α*′, *β*′).

Define F to be the flow field mapping from (*α*, *β*) to (*α*′, *β*′). Before any analysis and independently of any parameters, we calculate F to obtain a two-dimensional vector field evaluated over a two-dimensional grid of values *α*, *β*. In practice, instead of *α*, *β*, we evaluate over a grid of $l_{\mu }:=\log_{10}(\mu )$ and *l*_*C*_: = log _10_(*CV*), where *μ* = *α*/*β* and $CV=1/\sqrt{\alpha }$ are the mean and coefficient of variation of Γ(*α*, *β*), respectively. The grid is defined by 51 $l_{\mu }$ values equally spaced between −5 and 2 (i.e., log-spaced between *μ* = 10^−5^ and 10^2^) and by 50 *l*_*C*_ values equally spaced between −2 and 0 (CV between 10^−2^ and one). Units here are in coalescence time, where unit time corresponds to 2*N*_*e*_ generations. We exclude CV > 1 as this results in an infinite gamma density at *x* = 0. We make extensive use of the Arb library (Johansson 2017).

#### Segmentation and caching

Segregating sites are sparse, between long stretches of homozygosity. This can be used for “locus skipping,” performing a single step of the forward algorithm that accounts for a stretch of identical observation, based on a cached calculation. To this end, we calculate, for each element of the flow field grid and for each number of steps from one to a maximal cache size, the result of repeatedly applying the flow field and incorporating a hom observation. We calculate a similar set of values for stretches of missing values. The results (effectively two new flow fields, per number of steps) are cached and used in the forward pass. Whereas the flow field calculation step has to be performed once and independently of the scaled mutation and recombination rates, the caching step depends on the rates and is thus performed as a preprocessing step.

#### Input and output

Gamma-SMC works on a VCF file. In addition, it optionally accepts a list of BED files, one per sample in the VCF; each BED file describes a mask of genomic intervals, outside which alleles are considered missing, for this sample. If a single mask is applicable for all samples, a single BED can be provided instead. The user also describes at which sites to infer the TMRCA posteriors: either at heterozygous sites or across a grid of evenly spaced positions or both. The input is then segmented as described in the main text.

Within each segment, we need to calculate how many base pairs are homozygous and how many are missing. For each pair of samples and for each segment, we calculate the total length of the intersection of corresponding two masks and the segment. Then, we treat the segment as if it is made of a stretch of missing observations followed by a stretch of homozygous observations. The user must also provide the scaled mutation rate *θ* and scaled recombination rate *ρ*. Alternatively, if *θ* is not provided, it is estimated from the data as the average heterozygosity across diploid pairs. The recombination-to-mutation rate ratio may be provided instead of *ρ*.

Gamma-SMC can be applied to a subset of haplotype pairs, for example, only within diploids (for unphased genomes) or within a subset of samples. We output the final posterior gamma approximation, for each pair of haplotypes, at each output position.

#### Forward pass

For each pair of haplotypes, we perform the forward pass: For each segment, we begin from the $\left(l_{\mu },l_{C}\right)$ parameters describing the gamma approximation to the forward density at the end of the previous segment (or (0, 0) for the first segment). We look up in the cache the result of applying the flow field on a stretch of missing values of the length we observe, and get a new $\left(l_{\mu },l_{C}\right)$ pair; we then look up in the cache the result of applying the flow field on a stretch of hom values of the length we see; and finally, we incorporate the observation at the end of the segment, as explained in the Supplemental Methods.

When looking up a pair of values in a flow field, if one of the $\left(l_{\mu },l_{C}\right)$ values lies beyond the grid range, it is clipped back to the grid boundaries. Another clipping may be performed to ensure the entropy of the posterior does not increase; for details, see Supplemental Methods. Otherwise, they are interpolated (bilinear interpolation) using the four nearest neighbors.

We record the result of the forward pass at the output positions; that is, for each site required by the user for TMRCA inference, we record the $\left(l_{\mu },l_{C}\right)$ we obtained from the forward pass, describing the gamma approximation to the forward density at that site.

#### Backward pass

Instead of running the CS-HMM equivalent of the backward algorithm, we reformulate the backward pass in terms of applying the forward pass on the reverse sequence, with an additional transition through the flow field (Supplemental Methods). We again record the gamma approximation of the backward pass at the output position. Finally, we combine the forward and backward densities: If Γ(*α*, *β*) approximates the forward density at a position and Γ(*α*′, *β*′) the backward, then the combined posterior density will be Γ(*α* + *α*′ − 1, *β* + *β*′ − 1) (Supplemental Methods).

### Accuracy simulation study

We simulated 22 human chromosomes, using msprime v1.0.2 (Baumdicker et al. 2022). We used an effective population size of *N*_*e*_ = 15,000 (30,000 haploids), mutation rate *μ* = 1.25 × 10^−8^, and a human recombination map for each chromosome. We used ASMC v1.2 in sequencing mode. For the out-of-Africa model, we used the OutOfAfrica_3G09 model (Gutenkunst et al. 2009) from stdpopsim (Adrion et al. 2020) and the HapMapII_GRCh38 genetic map (The International HapMap Consortium 2007).

Gamma-SMC was run on simulated and 1000 Genomes data with *θ* = 0.00075 and *ρ* = 0.0006, a cache of size 10 kbp, and output every 1-kbp sites.

### Selection simulation study

We simulated two sets of samples. First, we used the *cosi2* (Shlyakhter et al. 2014) simulator to generate 100 replicates of 100 haploid sequences of length 10 Mbp, where the central position (at 5 Mbp) has undergone a selection sweep with a selection coefficient *s* and a final sample allele frequency *f*, using a mutation rate of *μ* = 1.25 × 10^−8^ and recombination rate of *r* = 10^−8^, per base pair per generation, and under a demographic model of a constant population size of *N*_*e*_ = 15,000. We simulated such sets across *s* = 0, 0.00001, 0.0001, 0.0005, 0.00075, 0.001, 0.002, 0.003, 0.004, 0.005, 0.01 and *f* = 0.55, 0.7, 0.9. Note that with this mode of simulation, we cannot control the mutation age.

Second, we used the SLiM (Haller et al. 2019; Haller and Messer 2023) simulator (v4.0.1) to generate 100 replicates of 100 diploid sequences of length 10 Mbp, where the central position (at 5 Mbp) has undergone a selection sweep with a selection coefficient *s*, beginning at age *g* (in generations), again using a mutation rate of *μ* = 1.25 × 10^−8^ and recombination rate of *r* = 10^−8^, per base pair per generation, and under a demographic model of a constant population size of *N*_*e*_ = 15,000. We simulated such sets across *s* = 0, 0.0001, 0.0005, 0.001, 0.005, 0.01 and *g* = 100, 500, 750, 1000, 2000, 3000, 4000, 5000. Note that with this mode of simulation, we cannot control the sample allele frequency.

On each set of sequences, we detected positive selection using iHS (Voight et al. 2006), nSL (Ferrer-Admetlla et al. 2014), Tajima's D (Tajima 1989), and the Gamma-SMC statistic we describe below. The iHS and nSL statistics were computed using the selscan (Szpiech 2022) software (v2.0) with default settings and normalized according to the sample allele frequency (25 bins) in neutral simulations (*s* = 0). These statistics were averaged across a window around the focal SNP; we experimented with window sizes of *W* = 100, 1 kbp, 10 kbp, or 100 kbp, or no window. Tajima's D was calculated across a window of either *W* = 50 kbp or 100 kbp, using the scikit-allel library (https://github.com/cggh/scikit-allel).

For Gamma-SMC, we inferred the pairwise posterior means every 1 kbp, using *θ* = 0.00075 and *ρ* = 0.0006. We then computed the proportion of posterior means below a threshold of *T* = 500, 1000, 2000, 3000, 4000, 5000, 10,000, 20,000, 50,000, 100,000, or 500,000 yr, with a generation time of 30 yr, and *N*_*e*_ = 15,000. We then averaged those proportions over a window of size *W* = 10 kbp or 100 kbp or no window around the focal SNP.

For each test, we calculate the TPR (the proportion of selected sites detected) and FPR (the proportion of neutral sites detected as selected), as a function of threshold on the statistic examined. From this, we calculated the AUC as a metric of the test's performance. We note that the recall is equivalent to TPR and that precision may not be a good metric in this case, as it is heavily affected by class imbalance (i.e., the proportion of loci under selection), so we do not report it here. If needed, the precision (and recall) can be calculated from the TPR, FPR, and the class proportions.

### Software availability

We implemented our method in C++, using vectorized SIMD operations. The code uses the Arb (Johansson 2017) and zstandard (https://facebook.github.io/zstd/) libraries. The code is available as Supplemental Code and at GitHub (https://www.github.com/regevs/gamma_smc).

## Supplementary Material

Supplement 1

Supplement 2

Supplement 3
