# Supplementary material for: Ultrafast genome-wide inference of pairwise coalescence times
Source: Genome Res. 2023 Jul;33(7):1023–31. doi: 10.1101/gr.277665.123 (PMC10538485; doi:10.1101/gr.277665.123)
Supplement: Supplement 2 [file Supplemental_Methods.pdf]

Ultra-fast genome-wide inference of pairwise coalescence times - Supplemental Methods

Regev Schweiger and Richard Durbin

Department of Genetics, University of Cambridge, Cambridge, United Kingdom

A Empirical study of gamma fits

To empirically examine how well a gamma distribution fits conditional posteriors, we conducted a simulation study. We simulated a single diploid genome (length of 10^8 basepairs, $\mu = 10^{-8}$ and $r = 10^{-8}$). We computed the forward pass of MSMC2 and recorded the forward conditional distributions. Then, for each one, we fit a gamma distribution by finding an (α, β) pair the minimizes the L2 distance. We observed a very good fit for most positions; we show 4 representative examples in Figure S1.

B The Sequentially Markovian Coalescent (SMC) model

We begin by describing the SMC. For a detailed overview, see e.g, the Supplement of (Wang et al., 2020). We denote by μ the unscaled mutation rate, in units of mutations per basepair per generation; by N_e the effective population size, in diploids; by $\theta = 4N_e\mu$ the scaled mutation rate; by r the unscaled recombination rate, in units of recombinations per basepair per generation; by $\rho = 4N_e r$ the scaled recombination rate; and by N the sequence length.

Hidden states and observations

An observation Y_i $(i = 1, \ldots, N)$ at position i in the genome for a pair of haplotypes, can take a value from $Y_i = y_i \in \{-1, 0, 1\}$, where -1 denotes missing data in either of the two haplotypes (not called, or masked out; see below), 0 denotes a site where both haplotypes have the same allele (i.e. a homozygous genotype in case of a single diploid genome), 1 denotes a mismatch between the alleles of the two haplotypes (i.e. a heterozygote genotype in case of a single diploid genome). Denote by $\mathbf{Y}_{1:i}$ the vector of random variables $(Y_1, \ldots, Y_i)$, and by $\mathbf{y}_{1:i}$ the vector of values they take $(y_1, \ldots, y_i)$.

The hidden states $\{X_i\}_{i=1}^N$ are the TMRCAs between the two alleles at position i. Each $X_i = x_i$ is given in units of coalescence time - where unit time corresponds to $2N_e$ generations. Similarly, denote $\mathbf{X}_{1:i} = (X_1, \ldots, X_i)$ and $\mathbf{x}_{1:i} = (x_1, \ldots, x_i)$.

Emission probability function

We use different emission probability functions conditional on the missingness patterns of the data. In positions where there is a missing observation, we skip the emission step, or equivalently define:

$$\Pr(Y_i = -1 \mid X_i = s) = 1$$

Otherwise, the emission probabilities for a given coalescence time s follow a Poisson distribution, with rate $\lambda = \theta s$. To see why, note that a coalescence time of s means there were $2N_e s$ generations until the coalescence of the two chains of ancestors. A mutation on either chain will lead to a het, so this is a Binomial distribution with $n = 2 \cdot 2N_e s$ and $p = \mu$. Since $n \gg 1, p \ll 1$, it can be well approximated with a Poisson distribution of rate $\lambda = np = 4N_e s\mu = \theta s$:

$$\Pr(Y_i = 0 \mid X_i = s) = e^{-\theta s}$$
$$\Pr(Y_i = 1 \mid X_i = s) = (\theta s) \cdot e^{-\theta s}$$

We assume $\theta \ll 1$, so that the probability of recurrent mutations is negligible.

Transition density function

The transition probability is derived from the SMC' model (Marjoram and Wall, 2006). A recombination event, which takes place at position i, detaches the chain of ancestors, which then coalesces back, either onto itself or onto the other branch, at time t. We note the SMC' model is a modification of the SMC model by McVean and Cardin (McVean and Cardin, 2005), that accounts for the case of the detached lineage coalescing back onto itself.

Formally, define:

$$q(t|s) := \Pr(X_{i+1} = t | X_i = s, R_i)$$

where R_i denotes the event of having a recombination event between position i and $i + 1$.

We assume a demographic model of a constant population size N_e. In this case, the probability of coalescing back onto the same branch is given by (Carmi et al., 2014), Eq (6), to be $(2t + e^{-2t} - 1)/4t$. The probability distribution of t assuming the detached lineage did not coalesce back onto itself is also given by (Carmi et al., 2014) to be

$$q_{\text{diff}}(t|s) = \begin{cases} \frac{1-e^{-2t}}{2s} & t \leq s, \\ \frac{e^{-(t-s)}-e^{-(t+s)}}{2s} & t > s. \end{cases}$$

This is combined together to give:

$$q(t|s) = q_{\text{diff}}(t|s) + \delta(t-s) \cdot \frac{2t + e^{-2t} - 1}{4t}$$

where δ is Dirac's delta function:

$$\delta(t-s) = \begin{cases} \infty & t = s, \\ 0 & t \neq s. \end{cases}$$

Also marginalizing on the event of recombination, we get

$$\begin{aligned} p(t|s) :=& \Pr(X_{i+1} = t | X_i = s) \\ =& \Pr(X_{i+1} = t | X_i = s, R_i) \cdot P(R_i | X_i = s) + \delta(t-s) \cdot P(\bar{R}_i | X_i = s) \end{aligned}$$

The probability of recombination can again be modelled as a Poisson with rate $\lambda = \rho s$ with a similar argument. Again, we assume $\rho \ll 1$, so we can neglect the event of more than one recombination. In this case, we choose to make an additional approximation and write:

$$\begin{aligned} \Pr(R_i | X_i = s) &= \rho s \\ \Pr(\bar{R}_i | X_i = s) &= 1 - \rho s. \end{aligned}$$

as this will simplify our calculations later.

C Posterior density in continuous-state HMMs (CS-HMMs)

The forward algorithm

The forward algorithm in the context of a CS-HMMs (Ainsleigh, 2001) is an iterative procedure which tracks the probability density (*forward density*) of a hidden state at a position i, given the observations until that position. We use the scaled variant (Bishop, Christopher M and Nasrabadi, Nasser M, 2006). Let $\hat{X}_i := X_i | (\mathbf{Y}_{1:i} = \mathbf{y}_{1:i})$. The forward densities are defined by:

$$\hat{\alpha}(x_i) := \Pr(\hat{X}_i = x_i) = \Pr(X_i = x_i | \mathbf{Y}_{1:i} = \mathbf{y}_{1:i})$$

with the recursion

$$\hat{\alpha}(x_i) = \frac{1}{c_i} \cdot \Pr(y_i | x_i) \int_0^\infty \Pr(x_i | x_{i-1}) \hat{\alpha}(x_{i-1}) dx_{i-1}.$$

with c_i a scaling factor that assures we get a normalized density function. We begin with an exponential prior $\Gamma(1,1)$, as this is the stationary distribution of the SMC' Markov chain.

Posterior state density

We use a slightly different alternative to the backward algorithm, which is easier to work with. Denote by $\overleftarrow{\hat{\alpha}}(x_i)$ the result of running the scaled forward algorithm on the reversed

sequence; that is,

$$\overleftarrow{\hat{\alpha}}(x_{i+1}) := \Pr(X_{i+1} = x_{i+1} | \mathbf{Y}_{i+1:N} = \mathbf{y}_{i+1:N})$$

Taking this one step back, we get:

$$\Pr(X_i = x_i | \mathbf{Y}_{i+1:N} = \mathbf{y}_{i+1:N}) = \int_0^\infty \Pr(x_i | x_{i+1}) \overleftarrow{\hat{\alpha}}(x_{i+1}) dx_{i+1}$$

Then, we have:

$$\begin{aligned}
\Pr(x_i | \mathbf{y}_{1:N}) &= \frac{\Pr(\mathbf{y}_{1:N} | x_i) \Pr(x_i)}{\Pr(\mathbf{y}_{1:N})} \\
&= \frac{\Pr(\mathbf{y}_{i+1:N} | x_i) \Pr(\mathbf{y}_{1:i} | x_i) \Pr(x_i)}{\Pr(\mathbf{y}_{1:N})} \\
&= \frac{\Pr(\mathbf{y}_{i+1:N} | x_i) \Pr(x_i | \mathbf{y}_{1:i}) \Pr(\mathbf{y}_{1:i})}{\Pr(\mathbf{y}_{1:N})} \\
&= \hat{\alpha}(x_i) \cdot \frac{\Pr(\mathbf{y}_{i+1:N} | x_i) \Pr(\mathbf{y}_{1:i})}{\Pr(\mathbf{y}_{1:N})} \\
&= \hat{\alpha}(x_i) \cdot \frac{\Pr(x_i | \mathbf{y}_{i+1:N}) \Pr(\mathbf{y}_{i+1:N}) \Pr(\mathbf{y}_{1:i})}{\Pr(x_i) \Pr(\mathbf{y}_{1:N})} \\
&= \hat{\alpha}(x_i) \cdot \int_0^\infty \Pr(x_i | x_{i+1}) \overleftarrow{\hat{\alpha}}(x_{i+1}) dx_{i+1} \cdot \frac{1}{\Pr(x_i)} \cdot \underbrace{\frac{\Pr(\mathbf{y}_{i+1:N}) \Pr(\mathbf{y}_{1:i})}{\Pr(\mathbf{y}_{1:N})}}_{Constant}
\end{aligned}$$

Namely, the posterior density of the TMRCA at a position i can be obtained by: (i) running the forward algorithm until step i; (ii) running the forward algorithm on the reversed sequence until step $i+1$; (iii) evaluating the integral one step back, to step i; (iv) dividing by the prior density of x_i; (v) scaling to obtain a legal density function.

D Gamma approximation

As mentioned above, assuming a standard coalescent process, with a constant population size, the coalescence time has an exponential prior; that is, $X_i \sim Exp(1) = \Gamma(1,1)$. Now, assume $\hat{X}_i \sim \Gamma(\alpha, \beta)$. We describe how to approximate $\hat{X}_{i+1}$ (or, more accurately, $\hat{\alpha}(x_{i+1})$).

Transition step

We give a closed-form expression for the coalescence time at the next step. We first use the approximation, assuming the recombination rate is small:

$$\Pr(R_i | X_i = s) = \rho s$$

Let $f_{\alpha,\beta}(x)$ be the probability density function (pdf) of the gamma function $\Gamma(\alpha, \beta)$. Assume that $\hat{X}_i \sim \Gamma(\alpha, \beta)$; then we define $p_{\alpha,\beta}(t)$ to be the compound distribution defined by the tran-

sition distribution conditional on $\hat{X}_i$:

$$p_{\alpha,\beta}(t) = \int p(t|s) \cdot f_{\alpha,\beta}(s) ds$$

Our goal is to approximate $p_{\alpha,\beta}(t)$ with a gamma distribution. We observe that empirically, with a small recombination rate, $p_{\alpha,\beta}$ is very close to $f_{\alpha,\beta}$. We therefore wish to find new gamma parameters (α' and β') that are small perturbations of the previous parameters (α and β):

$$\alpha' = \alpha + u \cdot \rho$$
$$\beta' = \beta + v \cdot \rho$$

Therefore, our goal is to find u, v that approximate $p_{\alpha,\beta}$ well. To this end, we wish to express $p_{\alpha,\beta}$ as a first-order Taylor approximation of $f_{\alpha,\beta}$ in terms of ρ. For this, we get:

$$\begin{aligned}
&\big(p_{\alpha,\beta}(t) - f_{\alpha,\beta}(t)\big)/\rho \\
&= e^{-t} \cdot \frac{(t\beta)^{\alpha}}{\Gamma(\alpha+1)} \left(M(\alpha, \alpha+1, -(\beta-1)t) - M(\alpha, \alpha+1, -(\beta+1)t)\right) \\
&+ \frac{2t + e^{-2t} - 1}{2} \cdot f_{\alpha,\beta}(t) + (1 - e^{-2t}) \cdot \frac{\Gamma(\alpha, \beta t)}{\Gamma(\alpha)} - 2t \cdot f_{\alpha,\beta}(t)
\end{aligned}$$

where M is Kummer's confluent hypergeometric function, also denoted ${}_1F_1$. The derivation is given in "Gamma-SMC transition step derivation" below.

PDE approach to gamma approximation

We can treat this last expression as the linear pertubration in the gamma pdf caused by a possible recombination. As such, it is natural to express it as a linear combination of the perburtations of $f_{\alpha,\beta}$ in its parameters α and β:

$$u \cdot \frac{\partial f_{\alpha,\beta}(x)}{\partial \alpha} + v \cdot \frac{\partial f_{\alpha,\beta}(x)}{\partial \beta} \approx (p - f)/\rho$$

Note that u, v vary with α, β. Then, we approximate

$$p_{\alpha,\beta}(x) \sim \Gamma(\alpha + \rho u, \beta + \rho v).$$

To find u and v, we first calculate the partial derivatives,

$$\begin{aligned}
\frac{\partial f_{\alpha,\beta}(x)}{\partial \alpha} &= f_{\alpha,\beta}(x) \cdot (-\psi^{(0)}(\alpha) + \log(\beta) + \log(x)) \\
\frac{\partial f_{\alpha,\beta}(x)}{\partial \beta} &= f_{\alpha,\beta}(x) \cdot (\alpha/\beta - x)
\end{aligned}$$

where $\psi^{(0)}$ is the digamma function. We would like to solve the least squares problem:

$$\arg\min_{u,v} ||u \cdot \frac{\partial f_{\alpha,\beta}(x)}{\partial \alpha} + v \cdot \frac{\partial f_{\alpha,\beta}(x)}{\partial \beta} - (p(x) - f(x))/\rho||^2.$$

To do so, we evaluate the partial derivatives, as well as $(p - f)$, over a grid of 2,000 values of x-s, placed to cover the main range of $f_{\alpha,\beta}$; then we solve:

$$\arg\min_{u,v} \sum_{i=1}^{2000} \left(u \cdot \frac{\partial f_{\alpha,\beta}(x_i)}{\partial \alpha} + v \cdot \frac{\partial f_{\alpha,\beta}(x_i)}{\partial \beta} - (p(x_i) - f(x_i))/\rho \right)^2.$$

We note that the last equation is in fact independent of all model parameters θ, ρ, N_e, and therefore this minimization problem can be performed once, independently of any specific parameters.

Log-coordinates

To better operate across scale, it is better to use coordinates specified in log-scale. If we use $\log_{10}(\alpha), \log_{10}(\beta)$ as our coordinates, by the chain rule, we get

$$\frac{\partial f_{\alpha,\beta}(x)}{\partial \log_{10}(\alpha)} = \frac{\partial f_{\alpha,\beta}(x)}{\partial \alpha} \cdot \frac{\partial \alpha}{\partial \log_{10}(\alpha)} = \frac{\partial f_{\alpha,\beta}(x)}{\partial \alpha} \cdot \frac{\alpha}{\log_{10}(e)}$$
$$\frac{\partial f_{\alpha,\beta}(x)}{\partial \log_{10}(\alpha)} = \frac{\partial f_{\alpha,\beta}(x)}{\partial \beta} \cdot \frac{\beta}{\log_{10}(e)}$$

Further, for improved interpretability and choice of grid boundaries, we wish to actually use the coordinates $\log_{10}(\mu), \log_{10}(C_v)$, where $\mu = \alpha/\beta$, $C_v = 1/\sqrt{\alpha}$. It follows that $\log_{10}(\mu) = \log_{10}(\alpha) - \log_{10}(\beta)$ and $\log_{10}(C_v) = -0.5 \cdot \log_{10}(\alpha)$. However, it turns out that those coordinates are not good for a Taylor expansion, so that it is not true that perturbation of gamma in these coordinates is approximately equal to a linear combination of the partial derivatives. Instead, to get the respective change in $\log_{10}(\mu), \log_{10}(C_v)$, we use $\log_{10}(\alpha), \log_{10}(\beta)$ and, using linearity, simply write that

$$\Delta \log_{10}(\mu) = \Delta \log_{10}(\alpha) - \Delta \log_{10}(\beta)$$
$$\Delta \log_{10}(C_v) = -0.5 \Delta \log_{10}(\alpha).$$

Emission step

Suppose we know (or approximate)

$$X_{i+1} | (\mathbf{Y}_{1:i} = \mathbf{y}_{1:i}) \sim \Gamma(\alpha, \beta)$$

Recall we use a Poisson emission model:

$$Y_{i+1}|(X_{i+1}=t) \sim Pois(\theta \cdot t)$$

Then, using the Markov property and Bayes rule,

$$\begin{aligned}
&\Pr(X_{i+1}=t|\mathbf{Y}_{1:i+1}=\mathbf{y}_{1:i+1})\\
&=\Pr(X_{i+1}=t|\mathbf{Y}_{1:i}=\mathbf{y}_{1:i}, Y_{i+1}=y_{i+1})\\
&=\frac{\Pr(Y_{i+1}=y_{i+1}|\mathbf{Y}_{1:i}=\mathbf{y}_{1:i}, X_{i+1}=t)\cdot\Pr(X_{i+1}=t|\mathbf{Y}_{1:i}=\mathbf{y}_{1:i})}{\Pr(Y_{i+1}=y_{i+1}|\mathbf{Y}_{1:i}=\mathbf{y}_{1:i})}\\
&=\frac{\Pr(Y_{i+1}=y_{i+1}|X_{i+1}=t)\cdot\Pr(X_{i+1}=t|\mathbf{Y}_{1:i}=\mathbf{y}_{1:i})}{\Pr(Y_{i+1}=y_{i+1}|\mathbf{Y}_{1:i}=\mathbf{y}_{1:i})}
\end{aligned}$$

In the numerator, $\Pr(Y_{i+1}=y_{i+1}|X_{i+1}=t)$ follows the Poisson distribution described above, and $\Pr(X_{i+1}=t|\mathbf{Y}_{1:i}=\mathbf{y}_{1:i})$ follows $\Gamma(\alpha,\beta)$. The denominator is constant. Therefore,

$$\begin{aligned}
&\Pr(X_{i+1}=t|\mathbf{Y}_{1:i+1}=\mathbf{y}_{1:i+1})\\
&\propto(\theta t)^{y_{i+1}}\cdot e^{-\theta t}\cdot\frac{\beta^\alpha}{\Gamma(\alpha)}\cdot t^{\alpha-1}e^{-\beta t}\\
&\propto t^{\alpha+y_{i+1}-1}\cdot e^{-(\theta+\beta)t}
\end{aligned}$$

and since this distribution must be normalized, we have that

$$\Pr(X_{i+1}=t|\mathbf{Y}_{1:i+1}=\mathbf{y}_{1:i+1})\sim\Gamma(\alpha+y_{i+1},\beta+\theta).$$

That is:

$$X_{i+1}|\mathbf{Y}_{1:i+1}=\mathbf{y}_{1:i+1}\sim\Gamma(\alpha,\beta+\theta)$$

if $Y_{i+1}=0$ (is homozygous), and

$$X_{i+1}|\mathbf{Y}_{1:i+1}=\mathbf{y}_{1:i+1}\sim\Gamma(\alpha+1,\beta+\theta)$$

if it is heterozygous. If Y_{i+1} is missing, we perform no updating.

Combining forward and backward passes

We have shown above that

$$\Pr(x_i|\mathbf{y}_{1:N})\propto\hat{\alpha}(x_i)\cdot\int_0^\infty\Pr(x_i|x_{i+1})\overleftarrow{\hat{\alpha}}(x_{i+1})dx_{i+1}\cdot\frac{1}{\Pr(x_i)}.$$

We wish to combine gamma approximations from both the forward and backward passes to obtain a gamma approximation to the full posterior. First, we run the forward pass with the

gamma approximation. This results in a gamma approximation to the forward density at each step, so that at position i we have $\hat{\alpha}(x_i) \sim \Gamma(a, b)$.

Second, we run the forward pass with the gamma approximation on the reversed sequence, so that at position $i+1$ we have $\overleftarrow{\hat{\alpha}}(x_{i+1}) \sim \Gamma(a'', b'')$. Then, we apply the flow field once, to represent the change in uncertainty after taking a single transition step (from $i+1$ to i). This results in a new approximation, $\int_0^\infty \Pr(x_i|x_{i+1}) \overleftarrow{\hat{\alpha}}(x_{i+1}) dx_{i+1} \sim \Gamma(a', b')$. We then have, at each step i, a gamma approximation for both $X_i|\mathbf{Y}_{1:i}$ and $X_i|\mathbf{Y}_{i+1:N}$. Then we get

$$\begin{aligned}\Pr(x_i|\mathbf{y}_{1:N}) &\propto \hat{\alpha}(x_i) \cdot \int_0^\infty \Pr(x_i|x_{i+1}) \overleftarrow{\hat{\alpha}}(x_{i+1}) dx_{i+1} \cdot \frac{1}{\Pr(x_i)} \\ &\propto x^{a-1} e^{-bx} \cdot x^{a'-1} e^{-b'x} / e^{-x} \\ &= x^{(a+a'-1)-1} e^{-(b+b'-1)}.\end{aligned}$$

As this function must normalize to a proper density function, it follows that (based on the previous gamma approximations)

$$X_i|\mathbf{Y}_{1:N} \sim \Gamma(a + a' - 1, b + b' - 1)$$

So, to combine both steps, we simply sum the α and β parameters, and subtract 1.

E Gamma-SMC transition step derivation

In this section we derive an explicit expression for $p_{\alpha,\beta}(t) - f_{\alpha,\beta}(t)\big)/\rho$. Recall that we approximate the probability of recombination, given a coalescene time of s, as:

$$\Pr(R_i|X_i = s) \approx \rho s$$

With this, the distribution of the next step is

$$\begin{aligned}p_{\alpha,\beta}(t) &= \int_{s=0}^\infty (\Pr(X_{i+1} = t|s, R_i) \Pr(R_i|s) + \Pr(X_{i+1} = t|s, \bar{R}_i) \Pr(\bar{R}_i|s)) \Pr(X_i = s)\, ds \\ &\approx \int_{s=0}^\infty [q(t|s) \cdot 2\rho s + \delta(t-s) \cdot (1 - 2\rho s)]\, f_{\alpha,\beta}(s)\, ds \\ &= \int_{s=0}^\infty q(t|s) \cdot 2\rho s \cdot f_{\alpha,\beta}(s)\, ds + (1 - 2\rho t) f_{\alpha,\beta}(t)\end{aligned}$$

where we have used the property of Dirac's delta function that $\int \delta(x)f(x)dx = f(0)$. We get that the difference between consecutive positions is:

$$
\begin{aligned}
p_{\alpha,\beta}(t) - f_{\alpha,\beta}(t) &\approx \int_{s=0}^{\infty} q(t|s) \cdot 2\rho s \cdot f_{\alpha,\beta}(s)\, ds \, + (1-2\rho t) f_{\alpha,\beta}(t) - f_{\alpha,\beta}(t) \\
&= \rho \left[\underbrace{\int_{s=0}^{\infty} q(t|s) \cdot 2s \cdot f_{\alpha,\beta}(s)\, ds}_{(*)} - 2t \cdot f_{\alpha,\beta}(t) \right]
\end{aligned}
$$

We proceed to develop $(*)$. We need to recall the following facts about the lower and upper incomplete gamma functions.

Fact 1 *For all* $t, \alpha, \beta > 0$,

$$
\begin{aligned}
f_{\alpha,\beta}(x) &:= \frac{\beta^\alpha}{\Gamma(\alpha)} x^{\alpha-1} e^{-\beta x} \\
\gamma(s,x) &:= \int_0^x t^{s-1} e^{-t} dt \\
\Gamma(s,x) &:= \int_x^\infty t^{s-1} e^{-t} dt \\
\int_0^t f_{\alpha,\beta}(x) dx &= \frac{\gamma(\alpha, \beta t)}{\Gamma(\alpha)} \\
\int_t^\infty f_{\alpha,\beta}(x) dx &= \frac{\Gamma(\alpha, \beta t)}{\Gamma(\alpha)}
\end{aligned}
$$

Fact 2 *For all* β *(including non-positive) and for all* $t, \alpha > 0$,

$$
\int_0^t x^{\alpha-1} e^{-\beta x} dx = \frac{t^\alpha}{\alpha} \cdot M(\alpha, \alpha+1, -\beta t),
$$

where M *is Kummer's confluent hypergeometric function, also denoted* ${}_1F_1$*. This generalizes the lower incomplete gamma function.*

We evaluate the integral separately at $s < t, s = t$ and $s > t$. For $s < t$,

$$
\begin{aligned}
\int_{s=0}^{t} q(t|s) \cdot 2s \cdot f_{\alpha,\beta}(s)\, ds &= \int_{s=0}^{t} \frac{e^{-(t-s)} - e^{-(t+s)}}{2s} \cdot 2s \cdot f_{\alpha,\beta}(s)\, ds \\
&= e^{-t} \int_{s=0}^{t} (e^s - e^{-s}) \cdot f_{\alpha,\beta}(s)\, ds \\
&= e^{-t} \cdot \frac{(t\beta)^\alpha}{\Gamma(\alpha+1)} \left(M(\alpha, \alpha+1, -(\beta-1)t) - M(\alpha, \alpha+1, -(\beta+1)t) \right)
\end{aligned}
$$

For $s = t$, and using Dirac's delta function to indicate a point mass,

$$\begin{aligned}\delta(0) \cdot q(t|t) \cdot 2t \cdot f_{\alpha,\beta}(t) &= \delta(0) \cdot \frac{2t + e^{-2t} - 1}{4t} \cdot 2t \cdot f_{\alpha,\beta}(t) \\ &= \delta(0) \cdot \frac{2t + e^{-2t} - 1}{2} \cdot f_{\alpha,\beta}(t)\end{aligned}$$

For $s > t$,

$$\begin{aligned}\int_{s=t}^{\infty} q(t|s) \cdot 2s \cdot f_{\alpha,\beta}(s)\, ds &= \int_{s=t}^{\infty} \frac{1 - e^{-2t}}{2s} \cdot 2s \cdot f_{\alpha,\beta}(s)\, ds \\ &= \int_{s=t}^{\infty} (1 - e^{-2t}) \cdot f_{\alpha,\beta}(s)\, ds \\ &= (1 - e^{-2t}) \cdot \frac{\Gamma(\alpha, \beta t)}{\Gamma(\alpha)}\end{aligned}$$

To summarize,

$$\begin{aligned}&\big(p_{\alpha,\beta}(t) - f_{\alpha,\beta}(t)\big)/\rho \\ \approx\, & e^{-t} \cdot \frac{(t\beta)^{\alpha}}{\Gamma(\alpha + 1)} \left(M(\alpha, \alpha + 1, -(\beta - 1)t) - M(\alpha, \alpha + 1, -(\beta + 1)t)\right) \\ +\, & \frac{2t + e^{-2t} - 1}{2} \cdot f_{\alpha,\beta}(t) + (1 - e^{-2t}) \cdot \frac{\Gamma(\alpha, \beta t)}{\Gamma(\alpha)} - 2t \cdot f_{\alpha,\beta}(t).\end{aligned}$$

F Entropy clipping

Entropy limits

For random variables X, Y, it is known that $H(X|Y) \leq H(X)$, where H is Shannon's entropy. In the context of the HMM underlying the SMC framework, an example of this principle is that a sequence of observations reduces our uncertainty about an unobserved TMRCA:

$$H(X_i | \mathbf{Y}_{1:N} = \mathbf{y}_{1:N}) \leq H(X_i).$$

Here, $H(X_i)$ is the entropy of the TMRCA at position i, prior to seeing any observations - this is the prior, here taken to be the exponential; and $H(X_i | \mathbf{Y}_{1:N} = \mathbf{y}_{1:N})$ is the entropy of this TMRCA given the diploid sequence. This princple also holds, for example, for the sequence of observations only up until position i - so that the forward distributions will have less entropy than the prior.

However, the update rules used for the transition step of Gamma-SMC are approximate, and therefore do not mathematically guarantee a reduction in entropy. To avoid inconsistency in inference, we need to explictly enforce this. We do so by setting an upper bound on the differential entropy of the gamma distributions used in Gamma-SMC.

The differential entropy of the gamma distribution is:

$$h(\alpha, \beta) := \alpha - \ln\beta + \ln\Gamma(\alpha) + (1-\alpha)\psi(\alpha)$$

where ψ is the digamma function. For the exponential prior $Exp(1) = \Gamma(1,1)$, this entropy is $h(1,1) = 1$, which we therefore require as an upper bound for entropy. This in turn also translates into a constraint of the coordinates we actually use, $\log_{10}(\mu), \log_{10}(C_v)$.

When this bound is violated, we can get nonsensical results. Consider for example, when combining the forward and backward passes as described above, we would have obtained $\Gamma(1, 1/3)$ from both passes. While this is a legitimate distribution on its own, when combining both, we get $\Gamma(1, -1/3)$ which is outside the defined parameter range of $\beta > 0$. This inconsistency stems from $\Gamma(1, 1/3)$ having an entropy larger than 1, which should not have happened.

We now prove that this is avoided. We can show that the gamma differential entropy is monotone increasing in α, for positive α values. To see that:

$$\begin{aligned}
\frac{\partial}{\partial\alpha} h(\alpha,\beta) = &\ \frac{\partial}{\partial\alpha}(\alpha - \ln\beta + \ln\Gamma(\alpha) + (1-\alpha)\psi(\alpha)) \\
&= 1 + \frac{\partial}{\partial\alpha}\ln\Gamma(\alpha) + (1-\alpha)\frac{\partial}{\partial\alpha}\psi(\alpha) - \psi(\alpha) \\
&= 1 + \psi(\alpha) + (1-\alpha)\psi^{(1)}(\alpha) - \psi(\alpha) \\
&= 1 + (1-\alpha)\psi^{(1)}(\alpha)
\end{aligned}$$

It can be proven (not shown here) that this derivative is always positive, as required. Now, recall that we require $\alpha \geq 1$; therefore, using the monotonicty and assuming the required entropy bound,

$$1 - \ln\beta = h(1,\beta) \leq h(\alpha,\beta) \leq 1 \Rightarrow 1 \leq \beta$$

To conclude, we have shown that if the differential entropy of a gamma distribution is below the threshold of 1; and $\alpha \geq 1$ as we enforce already; then $\beta \geq 1$ as well, which guarantees that combining the forward and backward passes will result in a legal gamma distribution.

Implementing clipping

To make sure the differential entropy remains below 1, we clip values to be in the legitimate range. We choose to fix the mean of the gamma distribution while reducing the CV sufficiently. If we recast h in terms of l_μ, l_C, then it can be shown that for a fixed l_μ and for $l_C \leq 1$, $h(l_\mu, l_C)$ is monotone increasing in l_C. We therefore wish to find the maximal l_C for which $h(l_\mu, l_C) = 1$. Unfortunately, we don't have a closed-form expression for that. Instead, we perform the following: (i) over a grid of l_μ values, find the maximal l_C for which $h(l_\mu, l_C) = 1$ using bisection; (ii) interpolate over the grid for new values.

Supplemental Figures

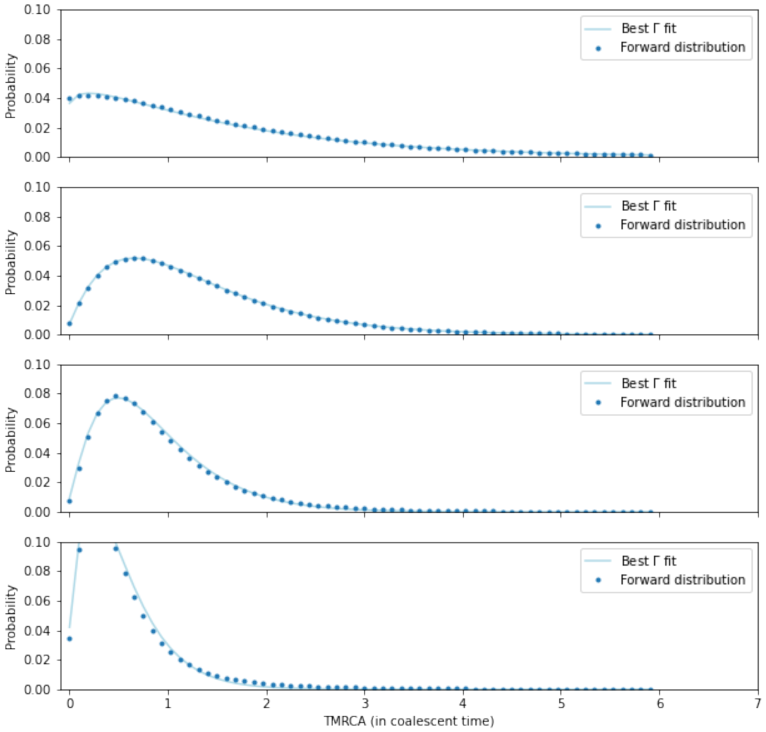

Figure S1: **Fitting gamma distributions.** Examples of best fit gamma distributions to forward conditional distributions, for four representive positions along a simulated genome.

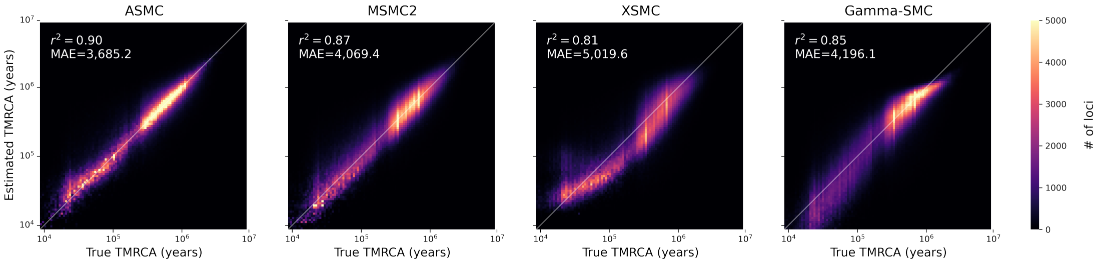

Figure S2: **Inference accuracy of ASMC-seq, MSMC2, XSMC and Gamma-SMC for out-of-Africa model.** A comparison of the true TMRCA vs. the estimated TMRCA (posterior mean) across a genome according to an out-of-Africa model. Pearson's r^2 and the mean absolute error (MAE, in generations) are shown.

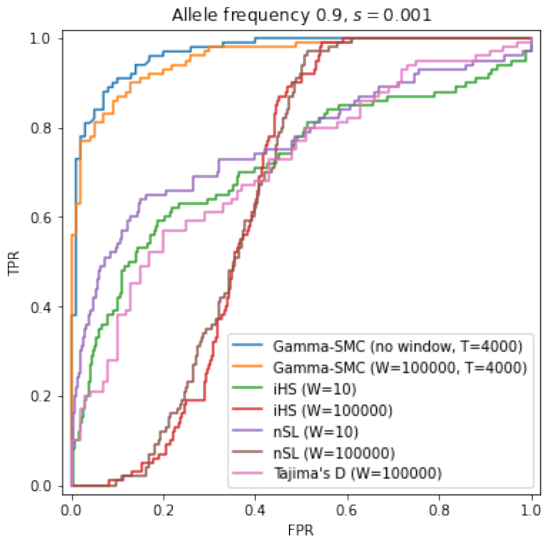

Figure S3: **Selection study.** A comparison of Gamma-SMC to alternative methods, with selection coefficient $s = 0.001$ and sample allele frequency 0.9.
